# 

**Published:** 1888-08

**Authors:** 


VOL. XVIII. AUGUST, 1888. NO. 8

### THE SOUTHERN MEDICAL RECORD.

LIBRARY INDEXED

EDITORS:

T. S. POWELL, M. D. R. C. WORD, M. D.

COLLABORATORS:

J. McF. Gaston, M. D.,
W. D. Bizzell, M. D.,
W. P. Nicolson, M. D.,
E. Van Goidtsnoven, M. D.,
P. R. Cortelyou, M. D.,
Julian J. Chisolm, M. D.
G G. Roy, M. D.,
A. G. Hobbs, M. D.,
W. S. Elkin, M. D.,
W. A. Crow, M. D.,
Jno. Thad Johnson, M. D.
K. P. Moore, M. D.

**Terms: $2.00 per Annum, in Advance; 4 Copies $6.00; Single Copies 20 Cents.**

Address R. C. WORD, M.D., Managing Editor, Atlanta, Ga.

Published and Entered as Second-class Matter at ATLANTA, GA.

